# Emergence of chikungunya seropositivity in healthy Malaysian adults residing in outbreak-free locations: Chikungunya seroprevalence results from the Malaysian Cohort

**DOI:** 10.1186/1471-2334-13-67

**Published:** 2013-02-05

**Authors:** Nor Azila Muhammad Azami, Sharifah Azura Salleh, Shamsul Azhar Shah, Hui-min Neoh, Zulhabri Othman, Syed Zulkifli Syed Zakaria, Rahman Jamal

**Affiliations:** 1 UKM Medical Molecular Biology Institute, Universiti Kebangsaan Malaysia, Kuala Lumpur, Malaysia; 2 Department of Medical Microbiology and Immunology, Universiti Kebangsaan Malaysia Medical Centre (UKMMC), Kuala Lumpur, Malaysia; 3 Department of Community Health, Universiti Kebangsaan Malaysia Medical Centre (UKMMC), Kuala Lumpur, Malaysia; 4 The Malaysian Cohort, Kuala Lumpur, Malaysia

**Keywords:** Adult, Malaysia/Epidemiology, Chikungunya virus, Seroprevalence study, Rural

## Abstract

**Background:**

In 1998, Malaysia experienced its first chikungunya virus (CHIKV) outbreak in the suburban areas followed by another two in 2006 (rural areas) and 2008 (urban areas), respectively. Nevertheless, there is still a lack of documented data regarding the magnitude of CHIKV exposure in the Malaysian population. The aim of this study was to determine the extent of chikungunya virus infection in healthy Malaysian adults residing in outbreak-free locations.

**Methods:**

A cross sectional study of chikungunya (CHIK) seroprevalence was carried out in 2009 amongst The Malaysian Cohort participants living in four states (Kuala Lumpur, Selangor, Pahang and Negeri Sembilan). A total of 945 participants were randomly identified for the study. Potential risk factors for CHIK infection were determined via questionnaires, and IgG antibodies against CHIK were detected by an enzyme-linked immunosorbent assay. Logistic regression identified risk factors associated with CHIK seropositivity, while geographical information system was used for visual and spatial analysis.

**Results:**

From the 945 serum samples tested, 5.9% was positive for CHIK IgG. Being male, Malay, rural occupancy and Negeri Sembilan residency were identified as univariate predictors for CHIK seropositivity, while multivariate analysis identified being male and rural occupancy as risk factors.

**Conclusions:**

This study provided evidence that CHIK is slowly emerging in Malaysia. Although the current baseline seroprevalence is low in this country, increasing number of CHIK cases reported to the Malaysia Ministry of Health imply the possibility of CHIK virus becoming endemic in Malaysia.

## Background

The Chikungunya virus (CHIKV) is a mosquito-borne virus of genus Alphavirus in the Togaviridae family which causes an acute viral infection characterized by fever, polyarthralgia, headache, and myalgia [1,2]. The name Chikungunya (CHIK) came from a Makonde language, which means ‘that which bend up’, describing the distinctive severity of joint pains due to CHIK infection [3]. *Aedes* (*Ae*.) *albopictus* and *Ae*. *aegypti* are the common vectors for this virus, and they are found both in rural and urban areas [4]. CHIK infections are usually initiated by bites of infected mosquitoes, from where CHIK fever develops after a 1–12 day incubation period [1]. Symptoms are usually self-limiting but could persist till day 10, although there were cases where the arthralgia and arthritis persist many months onwards [5]. Older patients continue to suffer joint pains and recurrent effusions for several years.

There are 3 genotypes of CHIK viruses namely the: West African, Asian, and Central/East African (which caused a worldwide outbreak in 2005) types. They are usually endemic in sub-Saharan Africa, India, Southeast Asia, Indonesia and Philippines [6,7]. The first outbreak of CHIK was reported in Mokande Plateau, Tanzania, where the virus was first isolated, and it later re-emerged in the Indian Ocean in 2005 [4,6,8]. The first appearance of CHIK in Southeast Asia was reported by Hammon; where it was isolated during the dengue and dengue hemorrhagic outbreak in Bangkok, Thailand, in 1960 [9].

Malaysia, with a population of approximately 28.3 million and a population density of 86 per square kilometre, has continuously recorded rising annual cases of CHIK infection since the first outbreak in this country in 1998 [10]. Nevertheless, national CHIK seroprevalence data is still lacking. Outbreaks of CHIK infection have been reported in Port Klang (1998), Bagan Pachor (2006), Ipoh, Perak (2006) and Johor (2008) [7,10,11]. High vector abundance and immigrant influx from endemic countries might have caused the outbreaks [10]. In the 1998 outbreak, females and those of Indian ethnicity were among those highly affected with a seroprevalence of 76.5% and 72.5%, respectively [10]. In the year 2008, 8320 patients with clinical diagnosis of CHIK were referred to the National Public Health Laboratory for laboratory confirmation and 3870 patients (46.5%) were positive [12].

To determine the extent and magnitude of CHIK infection in Malaysia, we conducted a pilot cross-sectional CHIK IgG seroepidemiology study beyond CHIK outbreak periods in healthy Malaysian adults living in the states of Pahang, Kuala Lumpur, Selangor and Negeri Sembilan of West Malaysia. The above states have not recorded any CHIK outbreaks before (Note: Port Klang is located at the western tip of Selangor, nevertheless in our current study, no samples from this area have been included). Geographic information system (GIS) was used to investigate whether seropositivities for CHIK were clustered within study areas.

## Methods

### Study population

Participants in this study were from The Malaysian Cohort (TMC) project which is a national project managed by our research institute (UKM Medical Molecular Biology Institute). This project was initiated in 2006 to recruit at least 100,000 Malaysians aged 35 and above, where these participants represent various ethnic groups, geographical locations and lifestyles. The TMC is a long term prospective study conducted to investigate the effects of gene, environment and lifestyle interactions in causing diseases. The project aims to identify risk factors and to discover new biomarkers for various diseases. Detailed information about each participant is collected along with blood, serum, plasma, lymphocytes and urine samples. All participants were without any acute illness during the time of sample collection and consented towards the storage and usage of their samples for medical and epidemiological research. Universiti Kebangsaan Malaysia Research Ethics Committee (UKMREC) had granted the ethical approval for TMC towards the use of human samples in this study.

### Sampling and sample size

**Figure 1:**
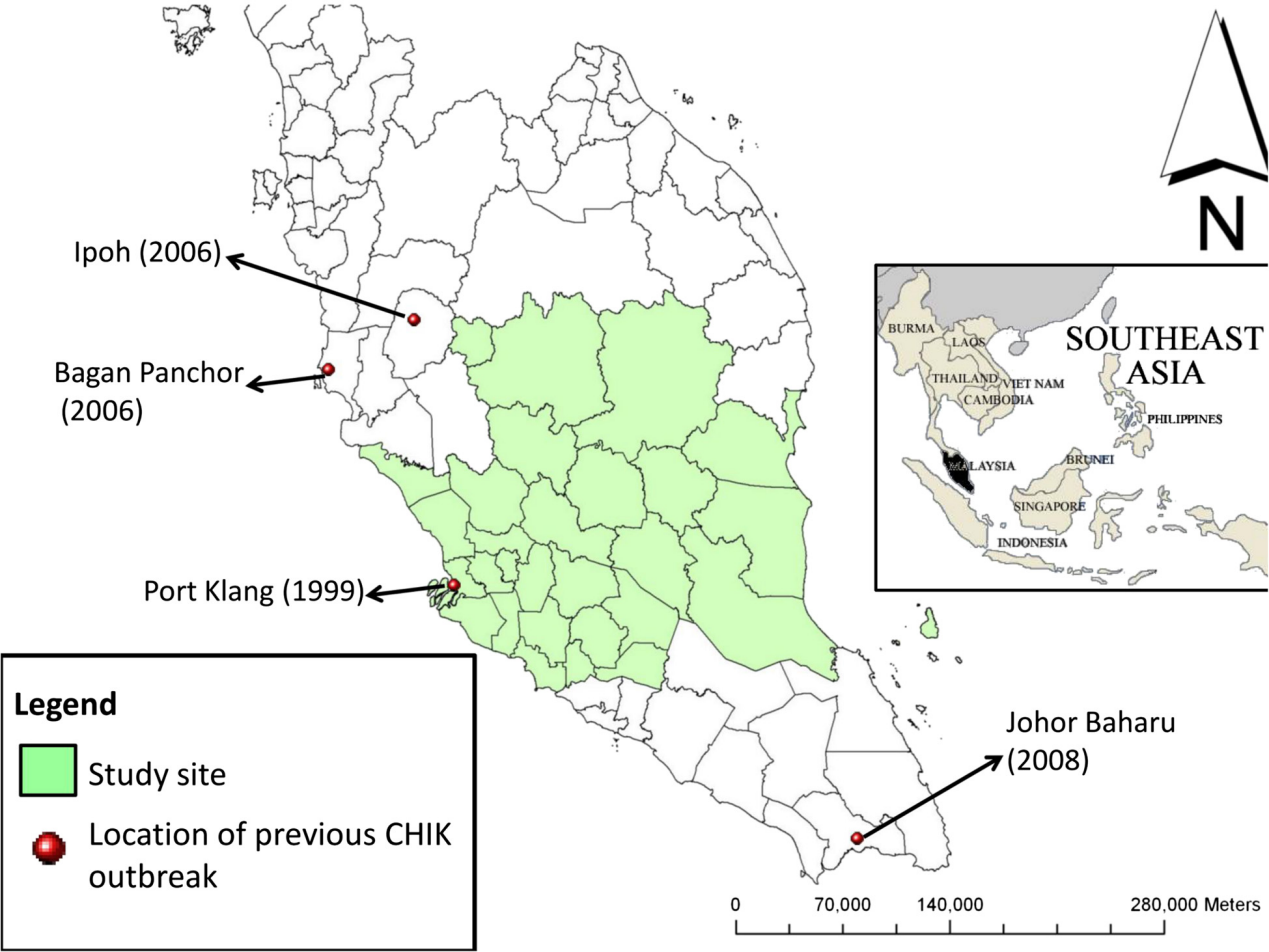
**Map of Peninsular Malaysia showing the study site** (**states of Negeri Sembilan**, **Pahang**, **Selangor and Kuala Lumpur**) **which is coloured in green. **Red buttons indicate previous CHIK outbreak locations. Small indicator map of South East Asia showing the location of Malaysia (coloured in black) is included.

A total of 13,330 participants were recruited into the TMC from 1 January 2008 to 31 December 2008. Sample size for this seroprevalence study was calculated under the assumption of 50% prevalence for CHIK infection with a ± 5% error using StatCalc Epi Info Version 6 software (CDC, Atlanta, USA). With this calculation, only 384 samples were required for this study; nevertheless, we randomly selected 945 from the initial population of 13,330 using the Minitab 15 software (Minitab Inc., Pennsylvania, USA), and their serum samples were retrieved for the study. Socio-demographic data of the participants such as gender, age, ethnicity, locality (urban/rural) were also retrieved from the TMC database. All serum samples were collected from CHIK outbreak-free locations (Figure 1).

### Serological testings

Blood samples (10 ml) were collected in Vacutainer® tubes (Becton Dickinson, Franklin Lakes, NJ), processed within 12 hours and stored at -80°C. Serum samples were measured for the presence of IgG antibodies to CHIK using NovaLisa Chikungunya Virus IgG capture ELISA kit (NovaTecImmundiagnostica GmbH, Germany). A positive result of CHIK IgG indicates previous exposure to CHIKV. Interpretation of ELISA results were done according to instructions from the manufacturer.

### Statistical analysis

Data analysis was performed using Statistical Package for Social Science (SPSS) 16.0 software (SPSS Inc., Chicago, USA). Differences in seropositivity between genders, age groups, states, ethnicities and localities were calculated by Chi-square tests and a *p* value of <0.05 was considered statistically significant. Logistic regression using the likelihood ratio method was used to identify risk factors associated with positive CHIK IgG. Statistically significant risk factors identified in the univariate analysis (*p*<0.05) were then included into the multivariate analysis. Odd ratios and their 95% confidence intervals were provided as estimates of the effect size and the test was conducted at 5% level of significance.

### Spatial analysis

To map CHIK prevalence, spatial location (coordinate) of each participant was first identified using Google Earth software (Google Inc. CA, USA). All coordinates were synchronized using World Geodetic System (WGS 84) which serves the (x, y) of an object by longitude and latitude, respectively. Spatial distribution and analyses of the CHIK seroprevalence were then managed and examined using ArcView GIS software version 9.3.1 (Environmental System Research Institute, Inc., Redland, CA). To describe CHIK distribution patterns, point pattern analysis using ‘Average Nearest Neighbour’ (ANN) tool of the software was used to determine the existence of significant clusters in the study area [13]. ANN measures the distance of each feature centriod and its neighbouring centriod locations, and calculates the average of entire “nearest neighbour distances”. The distribution is considered clustered if the average distance is less than the average of a hypothetical random distribution (R<1). However, if the average distance is greater, the distribution is considered as dispersed (R>1). Random distribution pattern is indicated by R =1.

## Results

Out of 945 participants, CHIK IgG was detected in 56 (5.93%) of them. CHIK IgG seroprevalence increased with age group, where participants aged 65–74 years old had the highest seroprevalence of 10.00% (5/50). Nevertheless, there were no significant differences of the seroprevalence between age groups (*p*=0.078). CHIK IgG seroprevalence according to ethnicity were as follows: Malay, 8.21% (42/517); Chinese, 3.25% (12/369); Indian and others 3.39% (2/59). [Note: In 2008, the ethnicity ratio of recruited subjects in the TMC was comparable to the national ratio of Malay: Chinese: Indian = 6:3:1]. Chi square analyses showed that CHIK IgG seroprevalence was significantly different between groups within the demographic factors of gender, state of residency, ethnicity, and locality (*p*<0.05) (Table 1).

**Table 1:** Characteristics and CHIK seropositivity of the 945 study participants

**Sociodemographic data**	**Total**^#^	**CHIK IgG seropositive**		**Chi square analysis**	
		**No**	**%**	**χ**^**2**^	**p**-**value**
Gender					
Male	376 (39.79%)	32	8.51	7.484	**0.007**^**§**^
Female	569 (60.21%)	24	2.54		
Age Range					
35–44	134 (14.18%)	3	2.24	6.830	0.078
45–54	450 (47.62%)	24	5.33		
55–64	311 (32.91%)	24	7.72		
65-74	50 (5.29%)	5	10.00		
Median Age, y (Range)	53 (35–74)				
Ethnicity					
Malay	517 (54.71%)	42	8.12	9.893	**0.007**^**§**^
Chinese	369 (49.53%)	12	3.25		
Indian & others	59 (6.24%)	2	3.39		
Locality					
Rural	386 (40.85%)	40	10.36	23.042	**<0.001**^**§**^
Urban	559 (59.15%)	16	2.86		
States					
Kuala Lumpur	275 (29.10%)	6	2.18	76.025	**<0.001**^**§**^
Selangor	211 (22.33%)	4	1.90		
Pahang	282 (29.84%)	11	3.90		
Negeri Sembilan	177 (18.73%)	35	19.77		

Table footnote:

^#^Except where indicated, values are number (%).

**Bold**^§^ indicates significant results, where *p*<0.05.

### Risk factor assessments

Logistic regression was performed to identify uni- and multivariate risk factors of CHIK seroprevalence. Gender, locality, ethnicity and state of residency were statistically significant univariate predictors of CHIK seroprevalence (Table 2). Males were more likely to be CHIK seropositive compared to females with an odds ratio of 2.112 (95% CI: 1.223- 3.647). Odds ratio for rural against urban occupancy was 3.923 (95% CI: 2.163- 7.115). Participants from Negeri Sembilan were more likely to be CHIK seropositive than participants from Kuala Lumpur (OR = 11.050, 95% CI: 4.540 -26.898) (*p*<0.001).

**Table 2:** Logistic regression to identify risk factors for CHIK seropositivity*

**Covariates**	**Unadjusted**			**Adjusted**		
	**OR**	**95% CI**	***p***-**value**	**OR**	**95% CI**	***p***-**value**
Gender			**0.007**^**§**^			**0.004**^**§**^
Female	1.000	-		1.000	-	
Male	2.112	(1.223, 3.647)	0.007	2.262	(1.299, 3.938	0.004
Locality			**<0.001**^**§**^			**<0.001**^**§**^
Urban	1.000	-		1.000	-	
Rural	3.923	(2.163, 7.115)	<0.001	4.088	(2.246, 7.439)	<0.001
Ethnicity			**0.010**^**§**^			**0.010**^**§**^
Indian & Others	1.000	-		1.000^¥^	-	
Malay	2.520	(0.594, 10.688)	0.210	2.520	(0.594, 10.688)	0.210
Chinese	0.958	(0.209, 4.393)	0.956	0.958	(0.209, 4.393)	0.956
State			**<0.001**^**§**^			**<0.001**^**§**^
Kuala Lumpur	1.000	-		1.000^¥^	-	
Selangor	0.866	(0.241, 3.110)	0.826	0.866	(0.241, 3.110)	0.826
Pahang	1.820	(0.664, 4.991)	0.245	1.820	(0.664, 4.991)	0.245
N.Sembilan	11.050	(4.540, 26.898)	**<0.001**^**§**^	11.050	(4.540, 26.898)	**<0.001**^**§**^

Table footnote:

*OR = odds ratio; CI = Confidence Interval.

¥ Not adjusted for other factors because there were no other significant individual factor.

**Bold**^§^ indicates significant values, where *p*<0.01.

Using multivariate analysis, only gender and locality were found to be significant risk factors for CHIK seropositivity (Table 2). The odds ratio for rural occupants was 4.088 (95% CI: 2.246- 7.439) compared to urbanites after adjustment for gender, while the male: female odds ratio was 2.262: 1.00 (95%CI: 1.299- 3.938) after adjustment for locality. Male participants from rural areas had the highest CHIK seropositivity rates (15.06%, 22/146) compared to female participants from rural areas (*p*= 0.24). However, urban males’ CHIK seroprevalence (4.35%, 10/230) was not significantly different compared to urban females (1.82%, 6/329) (*p* =0.119) (Table 3). In our study, participants from “urban” areas were those recruited from cities and town areas; while samples categorized as “rural” were collected from subjects living in villages and also settlers in plantations from the Federal Land Development Authority (FELDA) agricultural communities.

**Table 3:** Cross tabulation between gender and locality

**Covariates**	**Total**^#^	**CHIK IgG seropositive**^#^	***p***- **value**
Locality vs. Gender			
Rural Male	146 (15.45%)	22 (15.06%)	**0.024**^**§**^
Rural Female	240 (25.40%)	18 (7.5%)	
Total	386 (40.85%)		
Urban Male	230 (24.33%)	10 (4.35%)	0.119
Urban Female	329 (34.81%)	6 (1.82%)	
Total	559 (59.15%)		

Table footnote:

^#^Except where indicated, values are number (%).

**Bold**^§^ indicates significant results, where *p*<0.05.

### Geographical information system (GIS) analysis

**Figure 2:**
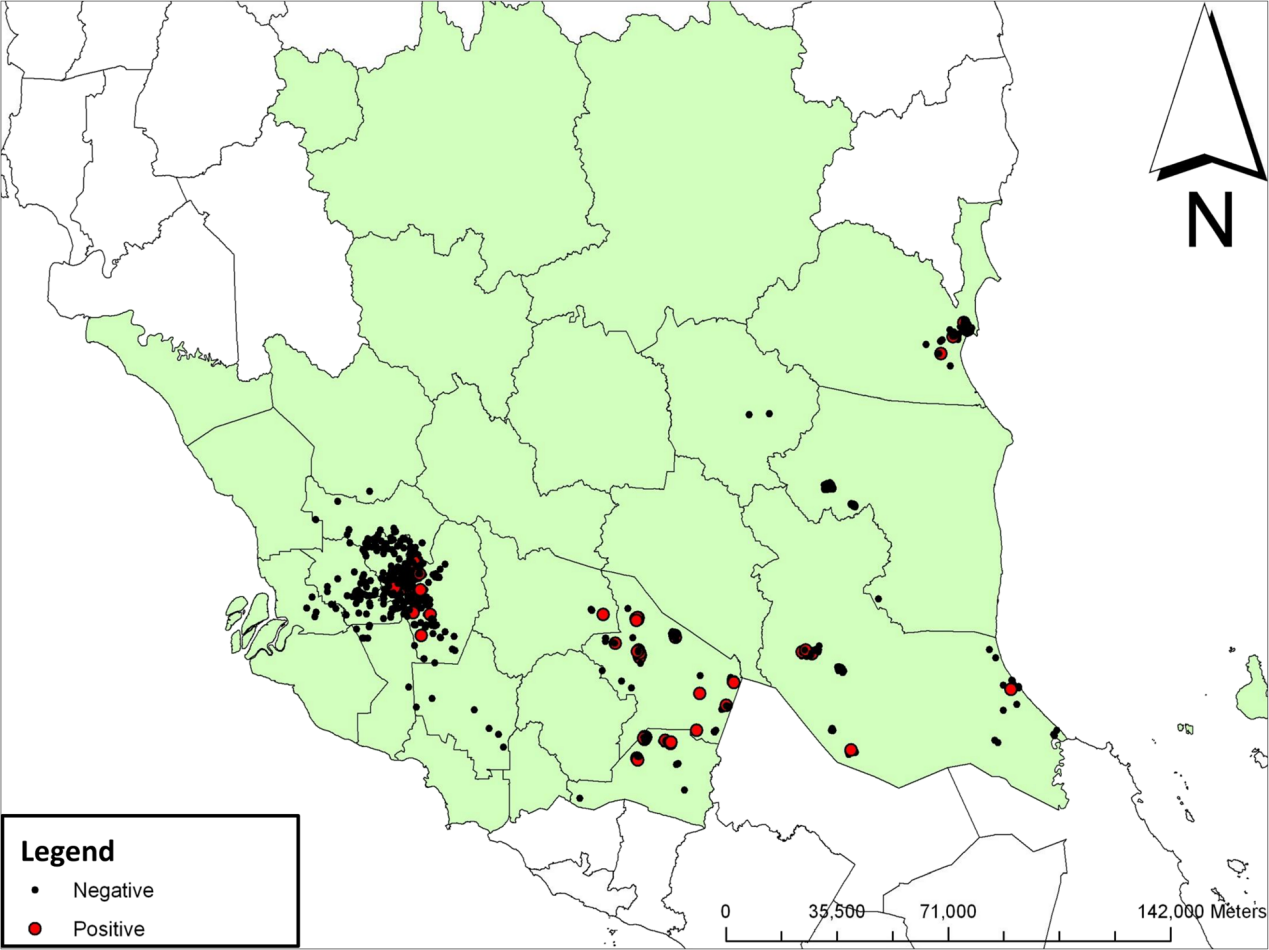
**Spatial distribution of CHIK seroprevalence in the study site. **Red dots represent chikungunya seropositive samples while black dots represent chikungunya seronegative samples. Lines define various districts in the states.

**Figure 3:**
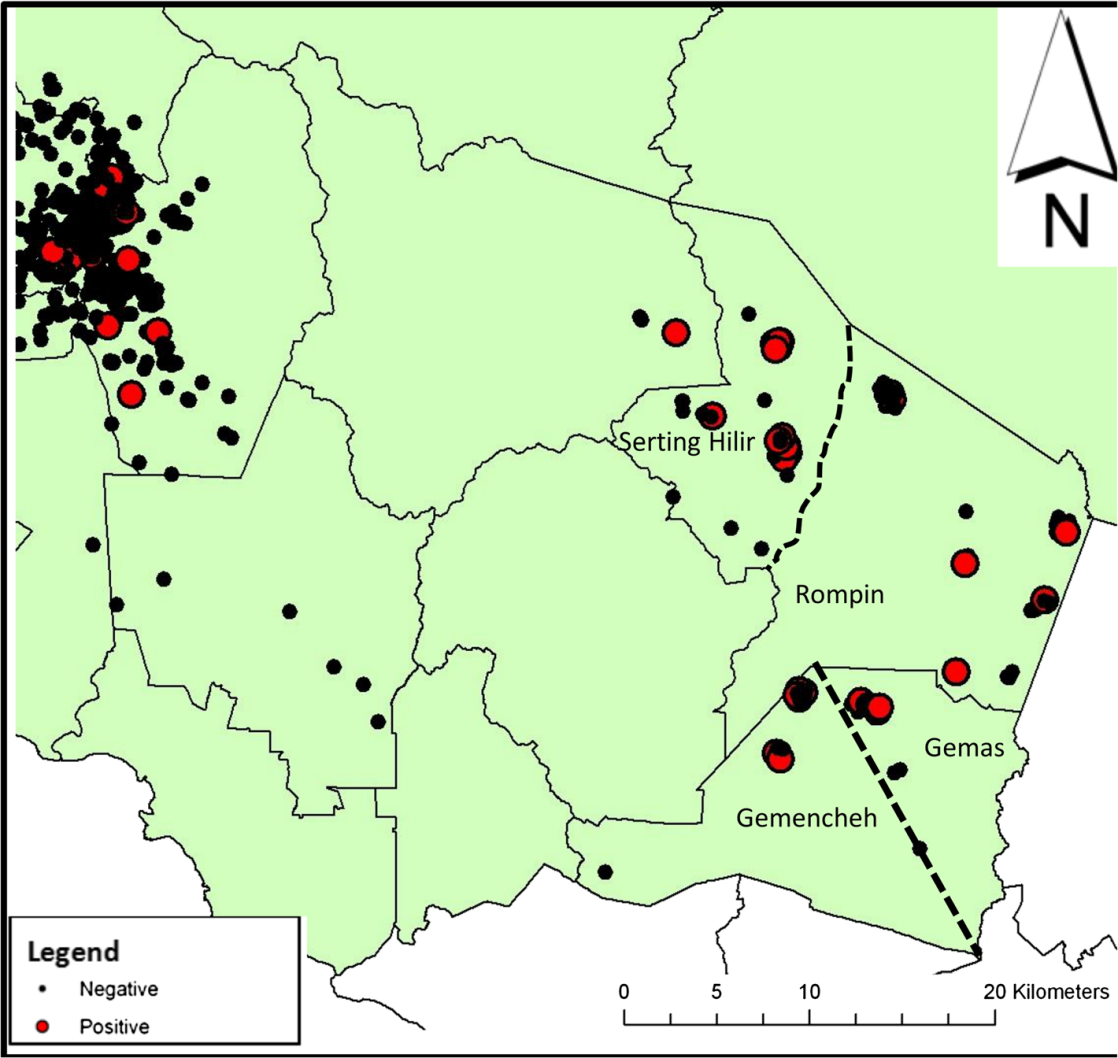
**Clustering of CHIK seroprevalence in the Negeri Sembilan state.** Red dots represent chikungunya seropositive samples while black dots represent chikungunya seronegative samples. Lines mark district borders while dotted lines define sub-districts.

Mapping of the participants’ residential addresses showed that CHIK seropositivity was distributed in all study areas (Figure 2). The distribution of CHIK seropositive subjects was found to be clustered (R=0.32, *p*<0.01) and several clusters were identified in the Negeri Sembilan (NS) state, where the participants were more likely to be CHIK seropositive compared to Kuala Lumpur (KL). The NS clusters were sparsely located compared to the more centralized clusters found in KL (Figure 3): two clusters were identified in both Serting Hilir (Jempol district) and in Gemencheh (Tampin district); while one cluster was identified in both Rompin (Jempol district) and Gemas (Tampin district). All other cases have been classified as sporadic cases.

## Discussion

Malaysia has a longer history of arbovirus surveillance compared to other Southeast Asian countries [14]. Nevertheless, as CHIK clinically resembles classical dengue fever, it is often misdiagnosed as dengue in dengue endemic countries like Malaysia [10]. Several dengue and haemorrhagic dengue epidemics have been reported in the 1970’s in Malaysia; however, no case of CHIK infection was reported during the same period [14,15].

Malaysia experienced its first CHIK outbreak in late 1998 which involved only the suburban community of Klang [12]. Seven years later, another outbreak emerged in a north-western coastal town of Perak, followed by outbreaks in Johor in 2008 [7,10-12]. Interestingly, our current cross sectional seroprevalence study conducted in CHIK outbreak-free locations of four West Malaysia states (Pahang, Selangor, Kuala Lumpur and Negeri Sembilan) showed that 5.9% of the study population has been exposed to CHIK infection. Nevertheless, this seroprevalence rate is still low compared to studies done in another location in Southeast Asia, which is the sub-district Bausasran located in the province of Yogjakarta. Similar to Malaysia, Bausasran recorded little to no CHIK seroprevalence [16]. However, in 1999, 33% of the study participants from Bausasran were reported to be CHIK IgG seropositive [16]. Yogjakarta itself experienced a few series of CHIK outbreak since 1983–1984, with the attack rate was estimated 70% -90% [16]. In 1999, cases of CHIK infection were reported at the Javanese city [16]. Results from the Yogjakarta and our studies imply that, although CHIK infections seemed comparatively lower in prevalence compared to dengue in Southeast Asia (such as 91.6% in Malaysia and 45% in Singapore among the adult population and 65.7% among the school children in Vietnam), sporadic outbreaks with high attack rates could still occur in this region [17-19]. Taking it all together, routine CHIK surveillance and vector control should be given due consideration in Southeast Asia and regions having tropical/sub-tropical weather that promotes the spread of CHIK vectors, to minimize and prevent the occurrence of outbreaks.

In our study, CHIK seroprevalence was found to increase with age, however the increase was not statistically significant (*p*= 0.078). Nevertheless, this finding is consistent with the results of a previous study in Emilia-Romagna Region (Italy) and Lamu (Kenya), where CHIK seroprevalence was found to be higher in older people [20,21]. The study in Yogjakarta also reported a similar pattern, where majority of the CHIK seropositives were found in individuals aged 15 years old and above [16].

As mentioned in the introduction, during the 1998 CHIK outbreak in Malaysia, females were reported to be highly affected with CHIK virus infection (76.5%) [10]. However in this study, males were found to have higher CHIK IgG seroprevalence of 8.51% (32/376), compared to 2.54% (24/569) for females, with a male:female odds ratio of 2.26: 1.00 (95% CI 1.299- 3.938) after adjustment for locality. In Mayotte, an archipelago located in the Indian Ocean, men also had higher CHIK seroprevalence compared to women, though the seroprevalence for both genders were much higher than that of our study (40.6% for men, 33.8% for women) [22]. An investigation carried out in neighbouring Comoros Island had contrasting results: they found that women were 1.7 times more likely to be infected compared to men [23]. The inconsistency of these findings might be related to specific behaviours, work places and community habits which were different between the two island nations in the Indian Ocean and possibly also in other countries where CHIK seroprevalence was investigated [22].

Our study showed that rural areas recorded higher CHIK IgG seroprevalence (10.36%) compared to the urban communities (2.86%). A serological study carried out in 1980 found that Malays who were living in rural areas had higher seroprevalence of CHIK antibodies, and monkeys were suggested to be important vertebrate hosts [24]. Our study also showed that participants who live in rural areas were 4.09 times (95% CI 2.246- 7.439) more likely to be CHIK IgG positive compared to those living in urban locations after adjusting for gender. Men who stay at rural areas had higher CHIK seroprevalence (15.06%) compared to urban males (4.35%). This phenomena might be due to the fact that most of our rural participants live in palm oil plantation settlements, in which the male participants would have a higher risk to be exposed to CHIK vectors due to plantation activities compared to urbanites. Nevertheless, about 2.86% of the urban population were found to be CHIK IgG positive in this study, even though a serological survey in 1980 had reported a complete absence of CHIK antibodies in urban Malaysian children and young adults [24]. In addition, prior to the 1998 outbreak, there was no evidence of CHIKV transmission in the urban areas of Malaysia [10].

GIS analysis showed clustering of CHIK IgG seropositivity in a few districts of Negeri Sembilan. All these clusters were situated in rural areas (palm oil plantation areas), where some local transmission might have occurred within the clusters. Nevertheless, the definite reason for the occurrence of these clusters in previously outbreak-free areas could not be determined in this study. We postulate that immigrant workers might have introduced CHIKV into these areas, as there were about 2 million migrant workers in Malaysia originating from neighbouring countries, including those in which CHIKV was endemic, in 2001 [10]. It is difficult to gauge the time CHIK emerged in this country due to the high dengue seroendemicity in its population, where misdiagnosis and under-reporting of CHIK infections were common due to mild hemorrhagic manifestations that have been described for both infections particularly among cases in Southeast Asia [6,17]. Human migration is also one of the plausible reasons to explain the emergence of CHIK in outbreak-free locations. Given the ease of transportation, populations from both outbreak and outbreak-free areas have become more mobile. Study participants from outbreak-free locations could have travelled to outbreak areas during the occurrence of the outbreaks and hence acquired CHIK infection. This is compounded by the fact that Malaysia is situated within contour lines of January and July isotherms, where CHIK vectors *Ae*. *aegypti and Ae*. *albopictus* mosquitoes survive and breed throughout the year, where constant transmission and exposure to the vector could facilitate the spreading of CHIKV.

This study has a few limitations. Data presented here was only a one-year data collected from Jan-Dec 2008 at CHIK outbreak-free locations, thus it does not represent the total prevalence rate of CHIK infection in Malaysia. Nevertheless, the results show that CHIKV is emerging in Malaysia, which necessitates prevention programmes in outbreak-free locations to prevent another major CHIKV outbreak. Our study also lacks human migration data which could help determine the cause and emergence of CHIK seropositive clusters in outbreak-free areas.

Due to the lack of validated ELISA assays for CHIK, positive ELISA results might be due to the presence of antibodies towards either CHIKV or O’nyong-nyong virus (ONNV), as both viruses belong to same genus and are highly related [25]. However, as ONNV has never been isolated in Malaysia, positive ELISA results obtained in this study are more likely to be caused by CHIK IgG instead of O’nyong-nyong IgG [7].

## Conclusions

In conclusion, 5.9% of the Malaysian population residing in CHIK outbreak-free locations have been exposed to this virus. Although the current seroprevalence is low in this country, the increasing number of CHIK cases reported to the Ministry of Health of Malaysia implies the possibility of CHIKV becoming endemic in Malaysia. High vector abundance and improved modes of transportation could facilitate movement of viremic individuals from one place to another, spreading the virus to new locations. In addition, further influx of migrant workers from CHIK endemic countries could also potentiate widespread CHIK infections in the Malaysian population. Prevention programs such as public health education and vector control strategies will be of crucial importance to prevent future CHIK outbreaks.

## Competing interests

The authors declare they have no competing interests.

## Authors’ contributions

AA carried out the immunoassay, performed statistical analysis and drafted the manuscript. SA and SAS participated in the design of the study and data interpretation. HM participated in the design of the study and drafted the manuscript together with AA. ZO and SZ participated in the design of the study and coordination. RJ conceived the study and participated in the study design and coordination. All authors read and approved the final manuscript.

## Pre-publication history

The pre-publication history for this paper can be accessed here:

http://www.biomedcentral.com/1471-2334/13/67/prepub
